# Editorial: Application of spatial information technology in infectious disease surveillance

**DOI:** 10.3389/fpubh.2024.1435397

**Published:** 2024-06-20

**Authors:** Min Xu, Chunxiang Cao, Zhenglong Li, Lin Zhao

**Affiliations:** ^1^Key Laboratory of Remote Sensing and Digital Earth, Aerospace Information Research Institute, Chinese Academy of Sciences, Beijing, China; ^2^Geoinformation and Big Data Research Laboratory, Department of Geography, The Pennsylvania State University, University Park, PA, United States; ^3^School of Public Health, Institute of EcoHealth, Shandong University, Jinan, Shandong, China

**Keywords:** spatial information, remote sensing, infectious diseases, spatial location, Geographic Information System (GIS)

Spatial information technologies, including Remote Sensing (RS), Geographic Information System (GIS), and Global Positioning System (GPS), hold great potential for public health and epidemiology, particularly in the context of infectious disease surveillance and control, where prompt location of cases, rapid communication of information, and quick mapping of the epidemic's dynamics are vital. For example, spatial information technologies enable the acquisition of large-scale, multi-temporal, high-accuracy disease related environmental parameters such as vegetation, water, soil and air quality, facilitate the identification of primary factors affecting disease transmission, and provide scientific and technological support for monitoring, early warning, assessment, emergency rescue, and mitigation before, during, and after an infectious disease outbreak (1).

Currently, spatial information technology has been widely applied in the study of infectious diseases surveillance and prediction. Earth observation is levered for enhanced infectious diseases monitoring (2). Geospatial big data is used for measuring and evaluating the impact of the environmental changes on human health (3). Infectious disease mapping, predictive modeling, and early warning have become more convenient with GIS tools (4). Novel approaches are proposed in examining the spatial heterogeneity and spatial-temporal clustering patterns of infectious diseases. Innovative spatial methods and computing technologies are employed for geospatial big data analytics in the context of infectious diseases.

The Research Topic entitled “*Application of spatial information technology in infectious disease surveillance*” aims to capture recent research in infectious diseases surveillance and control based on spatial information technologies, offering a forum for researchers to communicate their findings, scientific interpretations, and opinions with topics ranging from mapping of infectious diseases distribution to analysis and early warning of infectious disease transmission. The goal is to highlight the advances that have been made in this research area in recent years, and progress toward how to best analyzing spatial and temporal trends, stratifying risk factors, and assessing resource allocation.

This Research Topic includes four articles that improve our understanding of the connections between spatial information technology and infectious disease surveillance. Ghatee et al. investigate the distribution and occurrence of cutaneous leishmaniasis based on the geo-climatic factors in Southeast Iran using GIS. Their findings indicate that urban setting, orchard and agriculture areas, and MinMAT (minimum annual temperature) were the most important determinants of the distribution of cutaneous leishmaniasis in the region of Bam in southeastern Iran. The high-risk zones for cutaneous leishmaniasis are cities/large villages, agricultural and orchard areas in lower altitudes and with warmer climates and lower rainfall and humidity. Zhi et al. examine the diverse spatial patterns of COVID-19 within Wuhan by analyzing early case data alongside urban infrastructure information based accurate spatial location data. They assess both local and global spatial risks linked to the epidemic through co-location analysis, use the tool of Geodetector to identify facilities displaying unique spatial risk characteristics and reveal factors contributing to heightened risk. The findings unveil a noticeable spatial distribution of COVID-19 in the city, notably influenced by road networks and functional zones. Aldossari and Salam trace the differentials in COVID-19 infections, recoveries, and deaths across Saudi Arabia depending upon various demographic and developmental dimensions and interactions. Their analysis build a generalized linear model based on Saudi Arabia Ministry of Health data by classifying administrative areas and spatial locations information. The results indicate that more than the broader administrative areas, smaller homogeneous geographic units and locations played prominent roles in increasing the spread of COVID-19 and thereby recoveries and mortality. Song et al. develop and evaluate the KODARI app to demonstrate the effectiveness of the app supporting epidemiological survey, determine the significant factors affecting the intention to use, and confirm the applicability of new mobile framework by considering the specificity of infectious diseases, which can provide health authorities with a low-cost tool to monitor epidemics in various spatial scale including cities, states, and even communities with help of the built-in GPS positioning function of the mobile phone. The articles rising from the topic highlight the necessity and importance of spatial information technologies to monitor the infectious diseases.

With the continuous improvement of spatial resolution, temporal resolution, and spectral resolution of remote sensing, the continuous improvement of satellite navigation technology positioning accuracy, and the continuous enhancement of spatial data management and processing functions of GIS systems, spatial information technology can provide stronger scientific support for the monitoring and prediction of infectious diseases.

## Author contributions

MX: Writing – original draft, Writing – review & editing. CC: Writing – original draft. ZL: Writing – original draft. LZ: Writing – review & editing.

## References

[1] Huang ZB Cao CX Xu M Yang XW. Impact of environmental exposure on chronic diseases in China and assessment of population health vulnerability. ISPRS Int J Geo-Inform. (2023) 12:155. 10.3390/ijgi12040155

[2] Xu M Cao CX Guo HY Chen YY Jia ZW. Exploring the association between infectious diarrheal diseases and sea surface temperatures — coastal areas of China, 2009–2018. China CDC Weekly. (2022) 4:126–9. 10.46234/ccdcw2022.023 35265391 PMC8886489

[3] Bryan L Landrigan P. PM2.5 pollution in Texas: a geospatial analysis of health impact functions. Front Public Health. (2023) 11:1286755. 10.3389/fpubh.2023.1286755 38106908 PMC10722416

[4] Xu M Cao CX Wang DC Kan B Zhang H Jia HC. Districtprediction of cholera risk in China based on environmental factors. Chinese Sci Bull. (2013) 58:2798–804. 10.1007/s11434-013-5776-4

